# A descriptive study of ciguatera fish poisoning in Cook Islands dogs and cats: Demographic, temporal, and spatial distribution of cases

**DOI:** 10.14202/vetworld.2020.10-20

**Published:** 2020-01-03

**Authors:** Michelle J. Gray

**Affiliations:** Master of Veterinary Medicine Program, School of Veterinary Science, Massey University, Palmerston North, New Zealand

**Keywords:** cats, ciguatera, Cook Islands, demographics, dogs, epidemiology

## Abstract

**Background and Aim:**

Ciguatera fish poisoning (CFP) is the most common form of seafood toxicosis reported in humans worldwide. Dogs and cats are also susceptible to CFP, but there is little published and much unknown about the condition in these species. This study aimed to document the demographics of canine and feline cases of CFP, to examine the temporal and spatial distribution of cases, and to compare the incidence of animal and human CFP in the Cook Islands.

**Materials and Methods:**

Six years of medical records from the Esther Honey Foundation Animal Clinic (the only veterinary clinic in the Cook Islands during the study period) were reviewed to identify cases of CFP. The study variables included the date of presentation, species, age, sex, neutering status, and village/locality.

**Results:**

A total of 246 cases of CFP were identified, comprising 165 dogs and 81 cats. The sexes were equally represented; however, within each sex, entire animals outnumbered those that had been desexed. Cases occurred year-round, with slightly higher numbers recorded in spring. Annual case numbers trended downward over the study period. Cases were documented in all regions of Rarotonga and also one outer island (Aitutaki). Fewer cases were seen in areas with a narrow (<200 m) fringing lagoon, compared with a wide (>400 m) lagoon.

**Conclusion:**

This study documented epidemiologic patterns of canine and feline CFP cases for the first time. Based on the results, further investigation is warranted to establish whether desexing has a protective effect against CFP.

## Introduction

Ciguatera fish poisoning (CFP) is a multisystem toxicosis that afflicts a number of species, including humans, dogs, and cats. Cases of canine and feline CFP have been described sporadically in literature [1-6]. The toxicosis has also been discussed in articles and books [7-13]. There were some early experimental studies conducted [14-19], but there have been no objective studies of the condition published since the 1980s.

CFP is caused by the ingestion of fish containing ciguatoxins. Fish are not inherently toxic but rather acquire toxicity through the food chain in coral reef ecosystems [20-22]. Bottom-dwelling dinoflagellates of the genus *Gambierdiscus* are the source of the CFP toxin [23,24]. Herbivorous fish become toxic after ingesting *Gambierdiscus* spp. [25,26]. Similarly, carnivorous fish become toxic after eating ciguatoxin containing herbivores [27].

CFP is a global phenomenon. Toxic *Gambierdiscus* spp. are found in warm waters of the Pacific, Indian and Atlantic Oceans, and the Caribbean Sea [28]. Human CFP occurs in a corresponding circumglobal belt between latitudes 35°N and 35°S [29]. It can be assumed that canine and feline CFP occurs throughout the same geographic region, however, to date, all of the published case reports [1-6], experimental studies [14-19], and general articles [7-13] have originated in the Pacific.

Within the endemic region, spatial and temporal patterns of *Gambierdiscus* spp. and ciguatoxin containing fish are difficult to predict. Because reef fish tend to stay within a defined home range, toxic food webs can exist in discrete areas. One reef can be affected while an adjacent site is “safe” [30,31]. This heterogeneous, site-specific distribution is further complicated by temporal fluctuations in *Gambierdiscus* abundance and toxicity [32]. Areas previously “safe” may become ciguateric, and vice versa, as environmental factors impact on *Gambierdiscus* populations [31,33].

Risk factors for CFP have been studied in people, but not in animals. On a population level, environmental processes including reef disturbances and climate cycles are thought to influence the spatial and temporal occurrence of CFP [34-36]. On an individual level, demographic characteristics such as low socioeconomic status, male gender, and age have been (inconsistently) associated with CFP in people [37-40]. Finally, there are vector related factors: The risk of toxicity is thought to be higher with certain types/species of fish, with certain parts of the fish (e.g., the viscera/and head), and with larger portion sizes [40,41].

This report is the first to examine the epidemiologic patterns of CFP in dogs and cats. There are no data currently available regarding the demographic characteristics of animals afflicted by CFP. The occurrence of CFP in dogs and cats has never been tracked over time. Spatial analysis of canine and feline CFP cases has never been attempted. Research into these topics is necessary to identify the risk factors for the toxicity and develop mitigation strategies.

This study aimed to document the demographics of canine and feline cases of CFP and to examine the temporal and spatial distribution of cases. A secondary objective was to compare the incidence of canine and feline CFP with the incidence of human CFP in the Cook Islands.

## Materials and Methods

### Ethical approval

This retrospective review of case records was deemed to not require ethics approval (Massey University).

### Study site

The study was conducted in the Cook Islands, a country in which CFP is endemic in the human population [42]. Several articles evidence that CFP occurs in Cook Islands dogs and cats as well as their owners [3,4,7,9]. Cases for this study originated from the Esther Honey Foundation Animal Clinic, which provided the only veterinary service in the Cook Islands from 1995 to 2017.

### Study design

This was a retrospective case series.

### Case selection

The paper medical records of the Esther Honey Foundation Animal Clinic were searched for eligible cases. At the time of the study, handwritten records from 2011 onward were available for review. Cases presenting in the 6-year period March 2011-February 2017 were considered for inclusion. Inclusion criteria were: (1) A presumptive diagnosis of CFP documented by the attending clinician; and (2) no other diagnosis established during the period of care.

### Data collection

Eligible patient files were scanned to portable document format and assigned a case identification number. Each patient file was searched to identify the variables of interest: Date of presentation, species, age, sex, neutering status, and village/locality. Data were collected using Epi-Info software (version 7.2.1.0, CDC, Atlanta, USA).

The age variable was assigned categorical values based on the following criteria:

- Juvenile: Age given as ≤12 months or animal referred to as a puppy or kitten
- Adult: Age given as >12 months and <8 years or animal referred to as an adult
- Senior: Age given as ≥8 years or animal referred to as senior, aged, or geriatric
- Unspecified: Insufficient detail in medical record to classify the case as juvenile, adult, or senior.

For owned animals, locality was based on the animal’s place of residence.

For strays, locality was based on the place they were found.

### Statistical analysis

**Figure-1:**
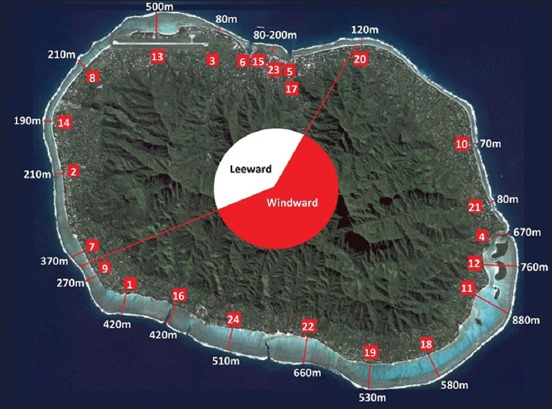
Rarotongan locations: Lagoon width and wind exposure. 1=Aroa, 2=Arorangi, 3=Atupa, 4=Avana, 5=Avarua, 6=Avatiu, 7=Betela, 8=Blackrock, 9=Kavera, 10=Matavera, 11=Muri, 12=Ngatangiia, 13=Nikao, 14=Ruaau, 15=Ruatonga, 16=Rutaki, 17=Takuvaine, 18=Tikioki, 19=Titikaveka, 20=Tupapa, 21=Turangi, 22=Turoa, 23=Tutakimoa, 24=Vaimaanga. Satellite image sourced from NASA [44].

Cases were automatically assigned lagoon width and wind exposure variables based on their locality (Table-1 and Figure-1) [35,43,44].

**Table-1:** Study locations: Assignation of geographic sector, lagoon width, and wind exposure.

Village/locality	Lagoon width^1^	Wind exposure^2^	Map reference (Figure-1)
Unspecified	Not applicable	Not applicable	-
Aitutaki	Not applicable	Not applicable	-
Aroa	Wide	Windward	1
Arorangi	Intermediate	Leeward	2
Atupa	Narrow	Leeward	3
Avana	Wide	Windward	4
Avarua	Narrow	Leeward	5
Avatiu	Narrow	Leeward	6
Betela	Intermediate	Leeward	7
Blackrock	Intermediate	Leeward	8
Kavera	Intermediate	Windward	9
Matavera	Narrow	Windward	10
Muri	Wide	Windward	11
Ngatangiia	Wide	Windward	12
Nikao	Wide	Leeward	13
Ruaau	Narrow	Leeward	14
Ruatonga	Narrow	Leeward	15
Rutaki	Wide	Windward	16
Takuvaine	Narrow	Leeward	17
Tikioki	Wide	Windward	18
Titikaveka	Wide	Windward	19
Tupapa	Narrow	Windward	20
Turangi	Narrow	Windward	21
Turoa	Wide	Windward	22
Tutakimoa	Narrow	Leeward	23
Vaimaanga	Wide	Windward	24

1 Lagoon width measured in Google earth, classifications based on those of Rongo and van Woesik [35]: Lagoon width <200 m=Narrow; 200 m<lagoon width <400 m=Intermediate; lagoon width >400 m=Wide.

2 Wind exposure based on the dominant south easterly wind direction [43] and consistent with that used by Rongo and van Woesik [35].

Descriptive statistics (frequency, mean, median, and range) were performed in Epi-Info.

Microsoft Excel was used to compare the temporal incidence of canine and feline CFP with that of human CFP in the Cook Islands.

## Results

Two hundred and forty-six cases with a presumptive diagnosis of CFP were identified from the 6-year pool of medical records. These comprised of 165 dogs and 81 cats.

Fifteen cases were excluded from the study. In these animals, CFP was listed initially as a differential, but alternate diagnoses were subsequently established (Table-2).

**Table-2:** Excluded cases: Final diagnoses.

Species	Excluding diagnosis
Cat	Aborting
Cat	Abscess
Cat	Chronic renal failure
Cat	Delayed organophosphate poisoning
Cat	Hemorrhagic gastroenteritis and shock
Cat	Hypoglycemia
Cat	Vestibular disease
Dog	Arthritis/hip pain
Dog	Arthritis/hip pain
Dog	Gastroenteritis
Dog	Intestinal parasitism
Dog	Respiratory disease
Dog	Respiratory disease/diaphragmatic hernia
Dog	Spinal injury/disc prolapse
Dog	Spinal injury/disc prolapse

### Demographics

#### Dogs

Females accounted for 49.1% of CFP cases (n=81) and males 50.9% (n=84). Table-3 presents a breakdown of cases of by age, sex, and neutering status.

**Table-3:** Age, sex, and neutering status of canine ciguatera fish poisoning cases.

Age	All dogs (n=165)				Females (n=81)				Males (n=84)			
		
	Entire	Desexed	Unspecified	Total	Entire	Desexed	Unspecified	Total	Entire	Desexed	Unspecified	Total
Juvenile^1^ (%)	19	3	6	28	21	5	4	30	18	1	8	27
Adult^2^(%)	6	7	1	15	7	5	0	12	5	10	2	17
Senior^3^ (%)	1	4	0	5	1	6	0	7	1	2	0	4
Unspecified^4^ (%)	20	21	11	52	22	20	9	51	18	21	13	52
Total (%)	47	35	18	100	52	36	12	100	42	35	24	100

1 Juvenile=Age given as ≤12 months or animal referred to as a puppy.

2 Adult=Age given as> 12 months and <8 years or animal referred to as an adult.

3 Senior=Age given as ≥8 years or animal referred to as senior, aged, or geriatric.

4 Unspecified: Insufficient detail in medical record to classify the case as juvenile, adult, or senior

#### Cats

Females accounted for 53.1% of CFP cases (n=43), males accounted for 39.5% (n=32), and the gender of 7.4% of cats was unspecified (n=6). Table-4 presents a breakdown of cases by age, sex, and neutering status.

**Table-4:** Age, sex, and neutering status of feline ciguatera fish poisoning cases.

Age	All cats (n=81)				Female cats (n=43)				Male cats (n=32)				Gender unspecified (n=6)			
			
	Entire	Desexed	Unspecified	Total	Entire	Desexed	Unspecified	Total	Entire	Desexed	Unspecified	Total	Entire	Desexed	Unspecified	Total
Juvenile^1^ (%)	16	1	1	19	16	2	0	19	13	0	0	13	33	0	17	50
Adult^2^ (%)	9	10	0	19	12	12	0	23	6	9	0	16	0	0	0	0
Senior^3^ (%)	0	0	1	1	0	0	0	0	0	0	0	0	0	0	17	17
Unspecified^4^ (%)	22	26	14	62	16	23	19	58	31	34	6	72	17	0	17	33
Total (%)	47	37	16	100	44	37	19	100	50	44	6	100	50	0	50	100

1 Juvenile=Age given as ≤12 months or animal referred to as a kitten.

2 Adult=Age given as >12 months and <8 years or animal referred to as an adult.

3 Senior=Age given as ≥8 years or animal referred to as senior, aged, or geriatric.

4 Unspecified: Insufficient detail in medical record to classify the case as juvenile, adult, or senior

### Temporal distribution

**Figure-2:**
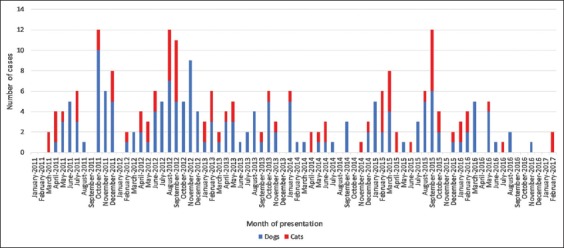
Cook Islands cases of canine and feline ciguatera fish poisoning (March 2011-February 2017).

Figure-2 depicts the occurrence of cases over the 6-year study period.

**Figure-3:**
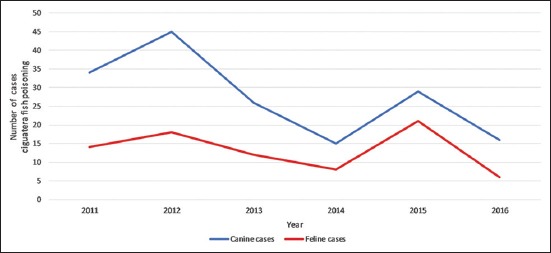
Number of cases of ciguatera fish poisoning by year and species.

**Figure-4:**
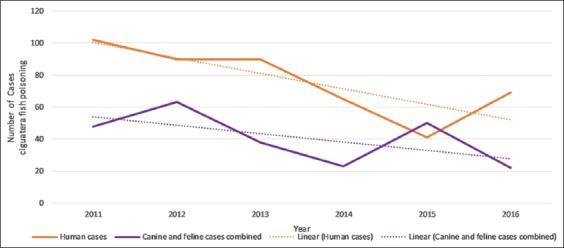
Number of cases of ciguatera fish poisoning by year: Comparison of animal cases and Cook Islands human [45] data.

An average of 41 cases of CFP was identified each year (range 22-63). Table-5 details the annual number of cases by species and also the number of human cases reported by the Cook Islands Ministry of Health over the same period. A comparison of canine and feline annual case numbers is presented in Figure-3 and a comparison of animal versus human case numbers in Figure-4 [45].

**Table-5:** Number of ciguatera fish poisoning cases by year; comparison with Cook Islands human CFP case numbers [45].

Year presented	Animal CFP cases				Cook Islands human CFP cases

	Canine cases	Feline cases	Total cases	%	
2011	34^1^	14^1^	48^1^	19.5	102
2012	45	18	63	25.6	90
2013	26	12	38	15.5	90
2014	15	8	23	9.4	65
2015	29	21	50	20.3	41
2016	16	6	22	8.9	69
2017	0^2^	2^2^	2^2^	0.8	No data
Total	165	81	246	100.0	457

1 Data from only 10 months of 2011.

2 Data from only 2 months of 2017, CFP=Ciguatera fish poisoning

**Figure-5:**
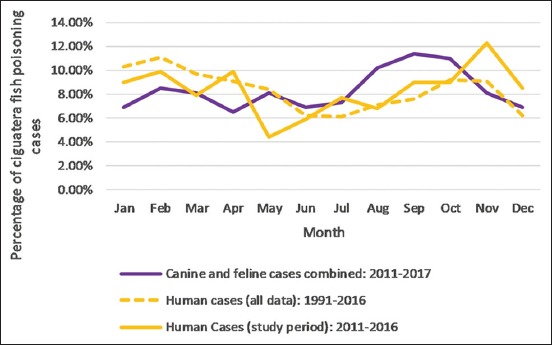
Percentage of ciguatera fish poisoning cases by month: Comparison of animal cases and Cook Islands human [45, 46] data.

Cases presented year-round, with a maximum of 12 cases seen in any 1 month. Case frequency by month is presented in Table-6. A breakdown of human CFP cases by month (as reported by the Cook Islands Ministry of Health) is included. A visual comparison is presented in Figure-5 [45-47].

**Table-6:** Canine and feline ciguatera fish poisoning cases by month; comparison with Cook Islands human CFP case numbers [45-47].

Month	Animal CFP cases	Cook Islands human CFP cases	
	
	2011-2017 cases (%)	2011-2016 cases (%)	1991-2016 cases (%)
January	17 (6.9)	41 (9.0)	485 (10.3)
February	21 (8.5)	45 (9.8)	522 (11.1)
March	20 (8.1)	36 (7.9)	456 (9.7)
April	16 (6.5)	45 (9.8)	428 (9.1)
May	20 (8.1)	20 (4.4)	397 (8.4)
June	17 (6.9)	27 (5.9)	292 (6.2)
July	18 (7.3)	35 (7.7)	286 (6.1)
August	25 (10.2)	31 (6.8)	334 (7.1)
September	28 (11.4)	41 (9.0)	356 (7.6)
October	27 (11.0)	41 (9.0)	434 (9.2)
November	20 (8.1)	56 (12.3)	429 (9.1)
December	17 (6.9)	39 (8.5)	290 (6.2)
Total	246 (100.0)	457 (100.0)	4709 (100.0

CFP=Ciguatera fish poisoning

### Spatial distribution

**Figure-6:**
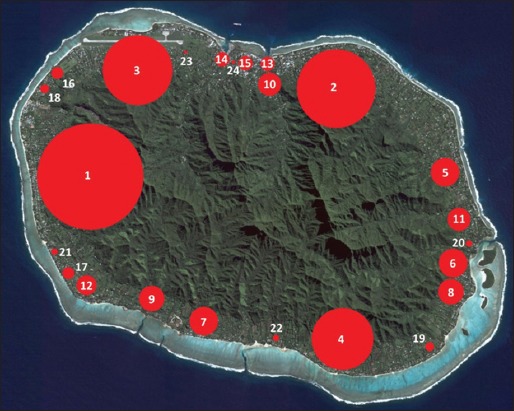
Geographic distribution of cases of canine and feline ciguatera fish poisoning in Rarotonga (March 2011-February 2017). Red circles indicate the approximate site of each locality; size of the circles is proportionate to the number of cases. Satellite image sourced from NASA [44].

Two hundred and twenty-three case records (90.6%) listed the animal’s village/district of origin. Twenty-four different localities around Rarotonga were specified, as well as one outer island (Aitutaki). The number of cases from each locality is reported in Table-7 and depicted in Figure-6 [44].

**Table-7:** Location of ciguatera fish poisoning cases.

Locality	Cases	Percentage	Map reference (Figure-6)
Arorangi	38	15.5	1
Tupapa	28	11.4	2
Nikao	25	10.2	3
Unspecified	23	9.4	-
Titikaveka	22	8.9	4
Matavera	10	4.1	5
Ngatangiia	10	4.1	6
Vaimaanga	10	4.1	7
Muri	9	3.7	8
Rutaki	9	3.7	9
Takuvaine	8	3.3	10
Turangi	8	3.3	11
Aroa	7	2.9	12
Avarua	5	2.0	13
Avatiu	5	2.0	14
Tutakimoa	5	2.0	15
Blackrock	4	1.6	16
Kavera	4	1.6	17
Ruaau	3	1.2	18
Tikioki	3	1.2	19
Aitutaki	2	0.8	-
Avana	2	0.8	20
Betela	2	0.8	21
Turoa	2	0.8	22
Atupa	1	0.4	23
Ruatonga	1	0.4	24
Total	246	100.0	

Further examination of case distribution was performed by grouping localities by lagoon width and the prevailing wind exposure. These results are presented in Table-8. The relative size of the human population in each region is included for comparison [35,43,48].

**Table-8:** Distribution of ciguatera fish poisoning cases by lagoon width and wind exposure; comparison with the resident human population.

Environmental criteria	Animal CFP cases^1^	% animal cases^1^	% human population^2^	Census localities included^2^
Lagoon width^3^				
Wide lagoon width >400 m	99	44.8	39.1	Nikao-Panama, Murienua, Titikaveka, Ngatangiia
Intermediate 200 m <lagoon width <400 m	48	21.7	18.1	Ruaau-Arerenga, Akaoa-Betela
Narrow lagoon width <200 m	74	33.5	42.9	KiiKii-Ooa-Pue, Tupapa-Maraerenga, Takuvaine, Tutakimoa-Teotue, Avatiu-Ruatonga-Atupa, Matavera
Wind exposure^4^				
Windward	124	56.1	51.2	KiiKii-Ooa-Pue, Tupapa-Maraerenga, Murienua, Titikaveka, Ngatangiia, Matavera
Leeward	97	43.9	48.8	Takuvaine, Tutakimoa-Teotue, Avatiu-Ruatonga-Atupa, Nikao-Panama, Ruaau-Arerenga, Akaoa-Betela

1 Data relate to 221 Rarotongan CFP cases (excludes 2 cases from Aitutaki and 23 with no location details).

2 Based on the 2011 census, figures for resident population [48].

3 Lagoon width measured in Google earth, classifications based on those of Rongo and van Woesik [35].

4 Wind exposure based on the dominant south easterly wind direction [43] and consistent with that used by Rongo and van Woesik [35]. CFP=Ciguatera fish poisoning

### Case clusters

Fifteen case clusters were identified, where multiple animals from the same locality were affected at the same time. Five clusters involved cats and ten involved dogs. Details on each cluster are provided in Table-9.

**Table-9:** Clusters of ciguatera fish poisoning cases.

Species involved	Number in cluster	Connection	Location	Dates of presentation (days ill)	
Cats	2	Same village	Tutakimoa	April 18, 2013	(12)
				April 18, 2013	(51)
Cats	2	Same household	Vaimaanga	August 24, 2013	(20)
				August 31, 2013	(22)
Cats	3	Same household	Ruaau	September 7, 2015	(21)
				September 9, 2015	(23)
				September 14, 2015	(14)
Cats	2	Same household	Betela	September 12, 2015	(3)
				September 12, 2015	(47)
Cats	2	Same household	Rutaki	February 26, 2017	(13)
				February 26, 2017	(13)
Dogs	2	Littermates, same household	Takuvaine	June 28, 2011	(3)
				June 28, 2011	(6)
Dogs	3	Littermates, same household	Arorangi	November 27, 2012	(2)
				November 27, 2012	(2)
				November 27, 2012	(12)
Dogs	2	Littermates, same household	Titikaveka	August 19, 2012	(2)
				August 19, 2012	(4)
Dogs	2	Same village	Vaimaanga	July 20, 2012	(7)
				July 21, 2012	(46)
Dogs	2	Same household	Arorangi	November 30, 2012	(5)
				November 30, 2012	(8)
Dogs	4	Littermates, same household	Tupapa	October 4, 2011	(5)
				October 4, 2011	(5)
				October 5, 2011	(2)
				October 5, 2011	(2)
Dogs	4	Same household	Kavera	September 21, 2015	(21)
				September 28, 2015	(10)
				September 30, 2015	(12)
				September 30, 2015	(23)
Dogs	4	Same household	Turangi	August 8, 2015	(3)
				August 8, 2015	(8)
				August 8, 2015	(24)
				August 8, 2015	(51)
Dogs	2	Same household	Turangi	March 13, 2016	(6)
				March 14, 2016	(11)
Dogs	2	Same household	Titikaveka	May 7, 2016	(22)
				May 10, 2016	(14)

## Discussion

### Study limitations

As a retrospective case series, this study has some inherent limitations. First, case file detail could not be standardized. Incomplete cases were still considered to contain potentially valuable information and were included in the study. Demographic variables (such as age and neutering status) were most frequently undocumented, and the amount of missing data needs to be considered when interpreting the results. Second, the methodology is unlikely to have captured all true cases of CFP that occurred on Rarotonga during the study period. Misdiagnoses and missed diagnoses are both possible and would both introduce inaccuracy into the analyses. The spatial analysis could also be distorted, if cases originating close to the clinic (located on the northwest of Rarotonga) were more likely to be presented than those living on the other side of the island. Finally, this study is limited by a lack of available data on the source population. Incidence rates cannot be calculated, and objective analysis of demographic and geographic risk factors is also impossible.

### Burden of disease

Two-hundred and forty-six cases were identified over the 6-year study period. This is more cases than have been documented in all previous case reports and experimental studies combined. The high number of cases may be a local anomaly. Recent literature does support the Cook Islands being a “hotspot” for ciguatera: The country had one of the highest annual incidence rates for human ciguatera in the Pacific (1998-2008), and lifetime prevalence rates in the resident population have been estimated at 52% [35,42]. Alternatively, the frequency of CFP seen in this study might indicate that CFP is a lot more common in cats and dogs than the sparse literature base suggests. The countries with the highest incidence of human CFP are small island nations. These countries often have limited veterinary services and produce few (if any) veterinary publications [49]. It is conceivable that dogs and cats in these countries could be regularly, or at least not uncommonly, afflicted by CFP without the wider veterinary community being aware.

It should be noted that the number of cases in this study almost certainly under-represents the true burden of disease. There are many mechanisms by which afflicted animals may have escaped the study population. Mild illness may not have been observed or considered to require veterinary attention; owners may have lacked transportation or have preferred the use of traditional remedies; animals may have been strays or simply ignored by their owner. Owner finances should not have precluded case presentation, as the Esther Honey Foundation is a charitable organization and does not charge for veterinary care. Elucidating the true burden of CFP in dogs and cats will require a well-designed cohort or cross-sectional investigation.

### Demographics

Demographic analysis of the study population found an approximately equal gender distribution, with entire animals outnumbering the desexed and juvenile cases equaling or exceeding adult cases. This does not, however, necessarily indicate differences in gender- or age-specific incidence rates. A lack of demographic data on the source population precludes the calculation of relative risks.

Further investigation is warranted, particularly to determine whether desexing does have a protective effect against CFP. A difference in incidence rates is conceivable, if entire animals spend more time roaming and scavenging, or if neutered animals are protected by a higher level of owner care and feeding. Human studies suggest that sex and youth are less likely to be risk factors for CFP. Regarding sex, reports have either found CFP to be gender independent [50,51] or have a slight bias toward males [37,39]. Glaziou and Martin [39] hypothesized that the latter situation is due to confounding (differences in fish consumption habits) rather than a true gender predilection. Regarding age, data from human CFP cohorts indicate a low incidence in children, with adults aged 30-49 being most frequently affected [38,39]^1^. The high incidence of juvenile cases in this study is most likely an artifact caused by the amount of missing data for the age variable. Less than half of the medical records specified the patient’s age. Logically, juveniles would be over-represented in the subgroup of cases with age data, given that their age is more easily recalled by owners and/or identified by veterinarians.

Note that breed was not included as a demographic variable because almost all dogs on Rarotonga are cross-bred “island dogs”, and cats would be predominantly characterized as domestic short-hairs. In the absence of any discernible variation, breed was not considered a useful parameter.

### Temporal distribution

This study documented high intermonth and interyear variation in CFP case numbers. Statistical testing of temporal trends was not attempted due to this high variability and the comparatively short study period.

Logically, the temporal incidence of CFP in dogs and cats should parallel human incidence rates, as all are exposed to ciguatoxins through the same food chain. The comparison of animal and human annual CFP case numbers (Figure-4) provides some support for this hypothesis. Linear trendlines for both groups showed a similar overall decline in CFP case numbers over the 6-years. The downward trend in CFP case numbers tallies with the work of Rongo and van Woesik [35,36]. They found CFP incidence on Rarotonga to be associated with positive phase of the Pacific Decadal Oscillation (PDO), *El Niño* events, and cyclone activity and predicted that shifting climate cycles would result in a decline in cyclone activity and CFP in the decade from 2010. Consistent with this prediction, there were no cyclones in Rarotonga during the study period [53]. Favorable climate phases did occur in the later stages of the study: Positive PDO in 2014-2017 [54] and strong *El Niño* in 2015-2016 [55]. However, as there is a lag period of 1-2 years before climate cycles influence CFP incidence [35,36], it is unsurprising that the overall trend in annual case numbers continued downward.

Similarities between animal and human CFP incidence were also found on a shorter time scale. Subjectively, it appears that both animal and human CFP case numbers are highest over spring/summer (Figure-5). CFP is generally described as non-seasonal [31,33]. However, in those locations where seasonality has been reported, the trend is for higher CFP incidence rates in the spring/summer [33,56]. No studies could be found that explicitly evaluate seasonal trends in human CFP in the Cook Islands, although monthly case numbers have been published by the Cook Islands Ministry of Health [46,47]. Further investigation is needed to establish if the incidence of CFP in the Cook Islands is truly seasonal.

### Spatial distribution

The spatial distribution of CFP cases in this study was not subjected to statistical testing. Without data on the geographic distribution of the source population, differences in incidence between localities could too easily be confounded by differences in local population size. Particularly, as some of the localities were large districts (e.g., Arorangi), while others were small villages (e.g., Turoa). Two of the cases originated from Aitutaki, one of the outer islands. Of the other outer islands, Atiu, Mitiaro, Mauke, Mangaia, Pukapuka, and Manihiki have all had cases of human CFP [57]. The lack of animal cases from these islands is likely due to difficulty in accessing veterinary care, rather than a true absence of CFP in the animal populations.

Given the limitations posed by a lack of data on the source population, a comparison of the spatial distribution of the human population and of animal CFP cases was performed (Table-8). Assuming that pet ownership rates are relatively uniform across the population, human population data could provide a surrogate measure of the geographic distribution of Rarotongan dogs and cats. The comparison suggests a relative paucity of CFP cases from localities with a narrow lagoon and from the leeward side of the island. These findings are plausible. In their survey of human CFP in the Cook Islands, Rongo and van Woesik [35] also found that areas where the lagoon is narrow had significantly fewer cases than areas with a wide lagoon. Although the same study found no significant differences between leeward and windward locations, wind exposure has been suggested by some as a risk factor for CFP [33,58]. In contrast, other studies have found *Gambierdiscus* spp. favor sheltered waters [21].

A weakness of this analysis (and indeed any geographic analyses of animal cases) is the risk that the ciguateric fish originated in a different locality to the animal. It is probable that in many cases, the fish were caught or bought elsewhere and transported home by the owner. This could confound attempts to associate environmental features of an animal’s location with the risk of ciguatera in the marine food chain.

## Conclusion

This article documented the demographics of animals afflicted by CFP in the Cook Islands and examined the temporal and spatial distribution of cases. The demographic results suggest a possible association between neutering status and CFP incidence. The temporal analysis found that the annual incidence was stable or declining over the study period, an observation that correlates with local reports of human CFP incidence. Case location data suggested a link between CFP incidence and geographic factors including lagoon width and wind exposure.

The epidemiologic patterns identified in this study need to be substantiated before any definite conclusions can be drawn. This will require the collection of demographic data on the canine and feline populations of Rarotonga through a census or cross-sectional survey. Comparisons could then be made between cases and non-cases to determine which variables are truly associated with CFP occurrence.

## Author’s Contributions

MJG was responsible for all parts of this project. The manuscript was written, edited, read, and approved by the author.

## Footnotes

1 Bagnis and Legrand [38] reported case distribution by age, when their data are adjusted for the population structure of French Polynesia in 1986 [52] incidence of CFP is highest in age groups 30-39 years and 40-49 years.
